# Meaning(s) of transition(s) from military to civilian life at the intersection with mental health: implications for clinical settings

**DOI:** 10.3389/fpsyg.2023.1142528

**Published:** 2023-05-09

**Authors:** Gabriela Misca, Jo Augustus, Jade Russell, Janet Walker

**Affiliations:** ^1^School of Psychology, University of Worcester, Worcester, United Kingdom; ^2^Institute of Health, University of Cumbria, Cumbria, United Kingdom; ^3^Institute of Health and Society, Newcastle University, Newcastle upon Tyne, United Kingdom

**Keywords:** veterans, transition, mental health, veteran clinical settings, PTSD, moral injury, connectedness

## Abstract

The experiences of military personnel moving into civilian life can be varied for the individual, families and communities. This paper aims to shed light on the various meanings of the multiple and “nested” transitions of military personnel to civilian life in the context of attending a mental health service in the UK. This was achieved through secondary analysis of semi-structured interviews with veterans who were engaging with a mental health service in the UK and a further interview with the mental health service lead. A thematic analysis was employed based on a descriptive phenomenological approach. Results indicate that an appropriate support infrastructure needs to be put in place for veterans prior to, during and after the transition to civilian life. The support appropriateness includes themes specific to connectedness to others, support service accessibility, mental health professionals' military culture awareness and mental health stigma. Although the findings suggest that community services need an awareness of veterans' specific needs, many of the themes are similar to those of the general population. Therefore, the need for an integrated healthcare system is essential in the transition of military service personnel to civilian life. Drawing on international evidence as well as the current findings, implications for policy and practice are highlighted throughout.

## 1. Introduction

Military personnel leaving active service, which may include combat related experiences, are faced with various challenges when returning and integrating into civilian life. These include, but are not confined to, mental health difficulties, some of which are linked to lengthy and multiple deployments during service to areas of conflict, although the presentation of these can occur a considerable time after deployment (Thompson et al., 2014). Moreover, the transitions out of active duty do not happen in a vacuum, notably in recent years as veterans return to struggling economies on the home front worldwide and their wide-reaching impacts on individuals, families and communities.

### 1.1. The UK and international context

In the UK there are 133,180 full time trained strength military personnel in the Army, RAF, Royal Marines and Royal Navy (MOD, 2022a). There has been a slight decrease (0.9%) in these numbers since 2021 (MOD, 2021). It is estimated that 10% of trained armed forces leave the military every year (Hynes and Thomas, 2016). This outflow or those leaving the military increased to 11.5% (*n* = 14,160), as reported in January 2022 (MOD, 2022b).

In recent years, the mental health and wellbeing of veterans and ex-service personnel have generated interest politically and, in the media, highlighting the need to develop services to support the transition and resettlement to civilian life of military veterans (Samele, 2013). Targeted health care for veterans has been traditionally in place in countries such as the USA, Canada and Australia via distinct administrative systems. However, in the UK, the responsibility of care for military personnel leaving the Armed Forces falls to the National Health Service (NHS) in the four nations of the United Kingdom, publicly funded and free at the point of delivery. Due to its generalist approach, mainstream NHS mental health services are often not well-equipped to respond to the needs of veterans. This is further compounded by the lack of tracking of the veteran status, although efforts are underway toward developing integrated electronic healthcare records of Armed Forces personnel. This, coupled with the stigma associated with mental health within military culture, results in a reticence to seek help and thus many veterans fall through the net of existing services (Britt, 2000; Vogt, 2011; Weeks et al., 2017; Britt et al., 2020).

Research in the UK (Iversen et al., 2005) indicates that after leaving military service the majority of ex-Service personnel have favorable outcomes; however there is a significant minority who will experience difficulties in this transition. Studies of UK military personnel indicated that common mental health problems such as depression, anxiety and alcohol misuse are most prevalent (Hoerster et al., 2012; Kaier et al., 2014; Koo et al., 2015). Rates of post-traumatic stress disorder (PTSD) in those who experienced deployment to Iraq and Afghanistan, and subsequently left the military are higher (9%) than those in the UK general population, with an estimated prevalence of PTSD and alcohol misuse of 4% (Stevelink et al., 2018; Mark et al., 2019). As a result, a significant minority of veterans will encounter emotional and mental health difficulties in transitioning to civilian life. There is a substantial body of research that highlights the concern about the levels of alcohol use within the UK Armed Forces (Iversen et al., 2007; Mark et al., 2019), however more research is needed into the effect of alcohol misuse among veterans and the impact on their families.

Military veterans will confront various obstacles in their return to civilian life; many will experience a lost sense of purpose, thus finding it harder to resume social activities; re-organizing family life will challenge both the veterans and their loved ones; and often many of these experiences will be associated with a common mental health problem, including anxiety, depression, alcohol misuse (Harvey et al., 2012; Kaier et al., 2014). Commonly, there is a corresponding reluctance to seek help due to a stigma in the military about seeking help (Iversen et al., 2011; Hoerster et al., 2012; Rafferty et al., 2018). Although it is important to note that there is also a stigma associated with seeking help for mental health difficulties, in the general population (Weeks et al., 2017). In addition, mental health services and support systems are not readily available, further compounding the difficulties of veterans experiencing difficult transitions. Therefore, mental health support and resources are critical to the personal and professional readjustment of military veterans and their families.

Whilst there is no Veteran Health Administration equivalent in the UK, however, due to the concerns highlighted above and pressure from veteran organizations, in 2010 the NHS invested in a pilot scheme of six treatment services aimed to meet the mental health needs of military veterans. These provided specialist service veterans and families outreach teams across the UK, establishing themselves as the source for regional veterans' mental health expertise, albeit an early evaluation of these services showed mixed results (Dent-Brown et al., 2010). However, despite such best efforts, veterans continue to experience significant barriers in accessing high-quality mental health care. It has been argued that one of the best resources is having access to mental health professionals who have combat experience and know first-hand the pressures of military and combat service. However, it remains unclear what professional skills are needed to treat military veterans, with research calling for a cross-cultural approach that looks at all aspects of functioning (Black et al., 2007).

There is a distinct gap in UK research about the impact of transitioning back to civilian life on veterans' families and the best practices for delivering mental health services to military veterans' and their families. There is need for further qualitative research to aid our understanding of veteran's experiences in relation to mental health. In addition, further exploration of the perceived stigma associated with transitional difficulties, as well as why some families are more resilient than others in making the reintegration to civilian life has been specially highlighted as a gap (Samele, 2013).

## 2. Perspectives on transition(s) from military to civilian life

There are multiple transitions associated with leaving the active military service, including the development of an identity as a civilian, (re)defining relationships within the family and community, and making decisions about work and personal life. These transitions do not happen in a vacuum, notably in recent years as veterans return to a struggling economy on the home front and its inherent negative impacts on individuals, families and communities. Furthermore, the effects of military service on the service member themselves influence their functioning and their ability to manage the transition from military service to civilian life. Transitions within and out of active military service have been examined from varied theoretical perspectives and some of these are highlighted below.

### 2.1. Homecoming theory

Undoubtedly, some the difficulties in transitions are linked to the features of the time spent in active service, such as the frequent and often lengthy deployments, which bring about disruptions to connections with family, friends and social networks (Charuvastra and Cloitre, 2008; Ahern et al., 2015; Williamson et al., 2021c). Homecoming theory (Schuetz, 1945) highlights the disconnection and alienation that can occur as a result of separation of one or more individuals (Adler et al., 2011; Koenig et al., 2014); and as such provides a framework to understand the barriers that service personnel, their family and friends can encounter in the transitions that occur both within and out of service. For example, during periods of separation such as deployments change occurs in both environments as a result of experiences encountered; thus “home” becomes unfamiliar when individuals return. Such unfamiliarity, coupled with different expectations, can lead to difficulties in family and friends reconnecting on their return home. Homecoming theory is therefore helpful to understand that veterans may be unfamiliar with aspects of their former lives. This is significantly different to the medical model, that seeks to assign a diagnosis of a mental illness and then offer appropriate treatments. However, this does not seek to help support the veterans' perception (Boscarino et al., 2022).

### 2.2. Resilience perspective

Through the lens of a context-dependent resilience perspective, the experiences of military veterans can be explained as the incongruity between strategies which promote resilience during active service but which may come into conflict with what resilience is seen in civilian and family life afterwards. For example, adherence to the hierarchy of military command underpins the resilience of military personnel in coping with life-threating situations whilst on duty; but the same quality may be less adaptive when manifesting within civilian systems, where for example, a strict adherence to established rules and hierarchies may not be effective in promoting harmonious flexible relationship functioning.

**Figure 1:**
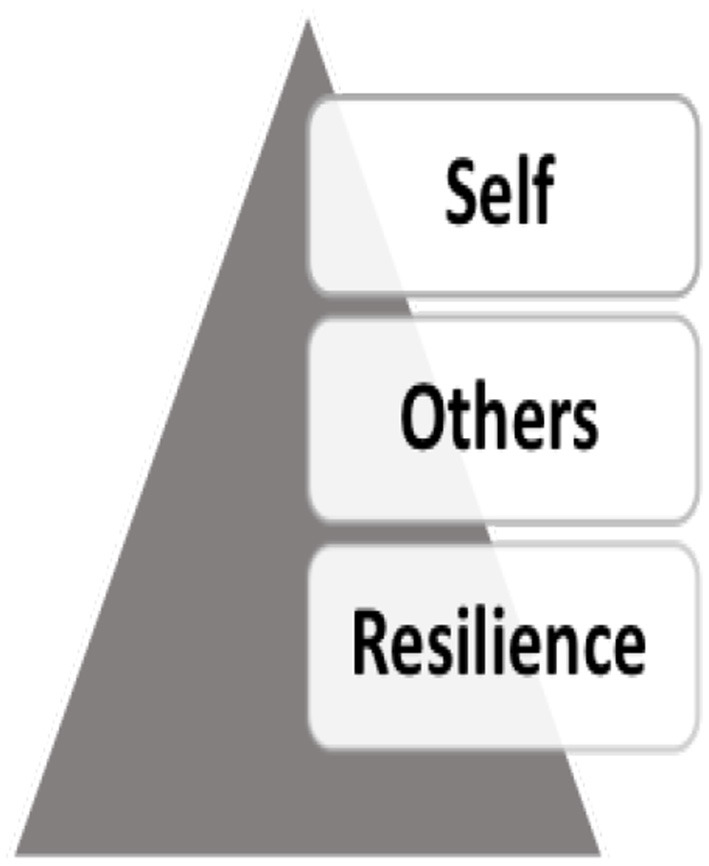
The perception of self and how others see them interacts with their sense of resilience.

In addition, research has explored the constructs that individuals hold in relation to transition into the community, highlighting how existing difficulties become more marked through transition, such as relational or co-morbidity (Hoge et al., 2007; Schnurr et al., 2009). Such issues could be innate or learned through exposure to military culture. Here Schlossberg's transition model is helpful to consider a strength-based approach in the factors surrounding transition (Schlossberg et al., 1995). There is a growing need to consider past and present psycho-social factors when considering the needs of veterans who transition to the community. Therefore, it is evident that there are a series of systems interacting; one's conscious sense of self; the perception of how others see them, and the individuals' own resilience, as illustrated in Figure 1.

Therefore, a successful transition in adapting to markedly different contexts requires flexibility and an ability to draw on strengths that are responsive to the immediate environment and its demands.

### 2.3. Cross-cultural transition

The military environment is a distinct role-based subculture that differs markedly from civilian life, with structure and rules, hierarchies and camaraderie being at the core of military life. Thus, central to an adaptive transition out of the military culture is the veteran's ability to navigate the rift between the military values and the culture and values of the civilian community, which often come in contrast with the military. In addition, the transition from active military service to home life places stress not only on the service member, but on their family member(s) as well, thus support networks may be affected (Black et al., 2007; Williamson et al., 2021c).

### 2.4. The meaning of transition(s) and the role of transition theory

While much of the research tends to focus on the role of physical and psychological injuries resulting from combat and non-combat situations, a critical intermediating variable within the transition process is the meaning that veterans attach to their situation and experiences. There is a lack of research into the inner experiences and associated constructs specific to the transition back to civilian life. By understanding more about the experiences, appropriate resources can be implemented to prevent the physical and psychological burden resulting from transitions. Therefore, this paper will explore the experiences of disruptions in connectedness of military service personnel, in order to support adjustment to such transitions.

Through the lens of Schlossberg's transition theory, the four factors; self, situation, support, and strategy, offers a framework to assess the transition of veterans from the military. An integrated model of transitions, that views transitions as “moving in” or “moving out” and encompasses learning processes in between (Goodman et al., 2006). Subsequently this theory can be applied to understand an individual's capability and challenges when making the transition to civilian life. This may include the identification of alternative coping strategies to overcome challenges.

Moreover, systematic research examining the broader issue of transition for former members of the UK armed forces entering the civilian world is lacking. Although, interventions have been designed to support successful transitions, they have often not been informed by systematic research. It is evident from existing research that an organized, supported transition, promotes the wellbeing factors of the transitional experience (Adler et al., 2011; Ahern et al., 2015). These experiences need to be analyzed further and are particularly relevant where an often-large military occupation is being withdrawn.

## 3. Study aims

In the context of these multiple and “nested” transitions of military personnel to civilian life, the objective of this paper is to explore the ways in which the veterans themselves understand their transition processes and the ways in which they derive meaning from their experiences. This paper also aims to address a notable gap in current research literature. This will be done through using a qualitative methodology to conduct a focused investigation of veterans' experiences of their own transition processes as they endeavor to make the transition from military to “civvy street”.

A qualitative approach was employed with this understudied population with the central aim of informing a richer understanding of these experiences in an effort to inform the development of support strategies and interventions. It is imperative to conduct research specifically targeting veterans to better understand and conceptualize their transitional experiences. Results of these studies can serve to establish appropriate services and inform helping professionals of how best to work with this population.

## 4. Methodology

This report is based on a secondary analysis of a qualitative data set collected as part of a service evaluation of veterans' engagement with and experience of a “Veterans Support Service” within a regional NHS Foundation Trust in England, UK (Misca, 2015). The veteran service had been in operation for over 6 years (at the time of evaluation) specializing in the assessment, treatment and intervention service for veterans coming into contact with mental health services in a region in central England. Treatment includes evidence based psychological therapies such as Cognitive Behavior Therapy (CBT) and Eye Movement Desensitization and Reprocessing (EMDR). The majority of service users are male, which does not entirely reflect the current serving population. In addition, the veterans on average had left the military 5 years prior to engaging with the veteran mental health service. The service makes between 80 and 250 contacts with service users per year.

The service evaluation study (which received ethical approval from the authors' University's Ethics Committee) explored the characteristics of veterans' service users accessing the service over a period of 3 months; their experiences of the service (i.e.,: veterans' perceptions of what worked for them and what did not) and their accounts of barriers to accessing and engaging with services. Unlike in the USA, in the UK there is no one specific mental health service for veterans, although in 2021 the NHS has launched “Op Courage” veterans' mental health service through which Armed forces veterans suffering a mental health crisis receive specialist care (NHS England, 2022). Nevertheless, it remains difficult to specify with certainty the number/percentage of veterans seeking or who have/sought mental health support services. Usually on entering civilian life, veterans and their families are expected to make use of the (free) NHS services, usually through primary care practitioners as a first point of contact and therefore veterans are not recognizable, unless they voluntarily disclose their veteran status. Although in the UK, the Armed Forces Covenant promotes fairness of and priority access to services where the condition relates to their service in the Armed Forces (MOD Defence Holistic Transition Policy, 2019). However, this relies solely on the individual identifying themselves as a veteran and, if they choose not to, the consequence could be a long waiting list (Iversen et al., 2011; Jones et al., 2013; Sharp et al., 2015).

The service evaluation included completion of a questionnaire and interviews with veteran service users attending the service. Six semi-structured interviews were conducted with veterans who were engaging with the mental health service; one further interview was conducted with the mental health service lead. The interviews with veterans which comprise the data set reported here offered rich data, as described by Charmaz (2006) as “data that is detailed, focused and full” (p. 14). The semi-structured interviews conducted as part of the evaluation study lasted ~40 min and elicited the veterans' views on accessing the veterans' support services, as well as gaining insight into the veteran's service background and transition, employment difficulties, family relationships, current difficulties, informal networks of support and their interests and involvement in the community. The interview with the service lead, a mental health professional and military reservist themself, explored similar areas from the perspective of service provider, such as views regarding veterans' perceived barriers to accessing mental health services, the socio-economic challenges faced by veterans, the impact of veterans' poor mental health on family members as well as veterans' integration into wider society (Williamson et al., 2021c). A thematic analysis was employed using a descriptive phenomenological approach (Braun and Clarke, 2006).

### 4.1. The analytical approach: descriptive phenomenological psychological method

A qualitative phenomenological research approach with explorative descriptive research designs was employed. A qualitative approach has been used to provide a detailed understanding of the veterans' perspectives and experiences. An explorative, descriptive research design method was used, to examine and gain new insight (De Vos et al., 2011; September and Beytell, 2019). Descriptive phenomenology is a research method that is widely used in social science as a method to explore “hidden” experiences (Matua and Van Der Wal, 2015). Phenomenology has been employed as a method of enquiry during the research process as a method to search for meaning, explore and describe the lived experience of individuals (Christensen et al., 2010). In the 6 phenomenological interviews from the existing set, data analysis focused on both the textural—e.g., the lived experience of the veteran—and the structural—e.g., the context in which it was experienced (September and Beytell, 2019).

### 4.2. Data analysis

Anonymised verbatim transcriptions of the interviews were re-analyzed (using the software package NVivo) for the purpose of this report within a thematic analysis framework with the specific aim to identify characteristics of key aspects of transition processes, identifying transition-related topics that arose repeatedly and considering how veterans explained these.

Two researchers independently read each transcript to identify important concepts and themes in the data. Initial analysis involved the creation of “open codes” that emerged from participants' narrative accounts, and subsequent stages of analysis involved inductive development of categories, subcategories, and themes. Similar attitudes or experiences are grouped in themes, reported by a number of participants and illustrated with quotes in the report. All quotes are completely anonymised and due to the sensitivity of the material, the identities of the participants were disguised in a manner that retains the essence of the individual narratives.

### 4.3. The participants

The six veteran participants represented a range of demographic and military careers characteristics: three were former military personnel in the Army, two veterans in the RAF, and one in the Navy. The age range of participants was 26–61, with a mean age of 46 years. Three participants were married or with a partner; two participants were divorced/separated, and one participant chose not to respond to the question. The presence of a partner may have an impact on the transition from military to civilian of the individual; partners can be used as a constant support through the process. However, this can also raise difficulties for partners of veterans to support the transition and cope with the possible mental health needs of the veteran.

Their average length of service among veterans was 11 years, ranging from 1 to 23 years. There was a broad range across participants in terms of time since leaving the service, ranging from 2 to 23 years. In the UK, a veteran is classed as someone who has served for more than 1 day in any branch of the armed services; they are entitled to hold the title of a veteran despite length of service, or years since leaving the service or type of leave from service.

Most participants left their military careers as their service was complete or voluntarily; two participants were given medical discharge and one participant's leave was a result of dishonorable discharge. The nature of the leave taken from the service may be of relevance because a person who is mentally prepared to leave such as service complete, may be more prepared and therefore their transition may feel more successful than a person whose leave is unexpected and out of their control. This implies that the type of discharge a veteran receives may go on to affect their future employment, which is linked closely with transition.

Moreover, research on UK personnel leaving the forces reported that mental health problems were more commonly reported among early service users (leaving before completing their 3–4.5 years minimum Service) than other service leavers, highlighting a potential need to target interventions to support their transition to civilian life and prevent the negative mental health outcomes experienced by ESLs further down the line (Buckman et al., 2012).

All participants had undergone operational deployments, between 1 and 7 times each and one participant had been on over 25 operational deployments. The link between operational deployments and mental health is disputed, with mixed evidence suggesting a link between mental health issues and operational deployments and/or direct experiences of conflict (Harvey et al., 2012).

## 5. Findings

The richness of data allowed for selective analysis focusing specifically on three overarching themes depicting “barriers to becoming a civilian”:

A. Intersection between transition and mental health
B. Past interfering into present—or “the military baggage”
C. Barriers to “civvy street” integration

**Figure 2:**
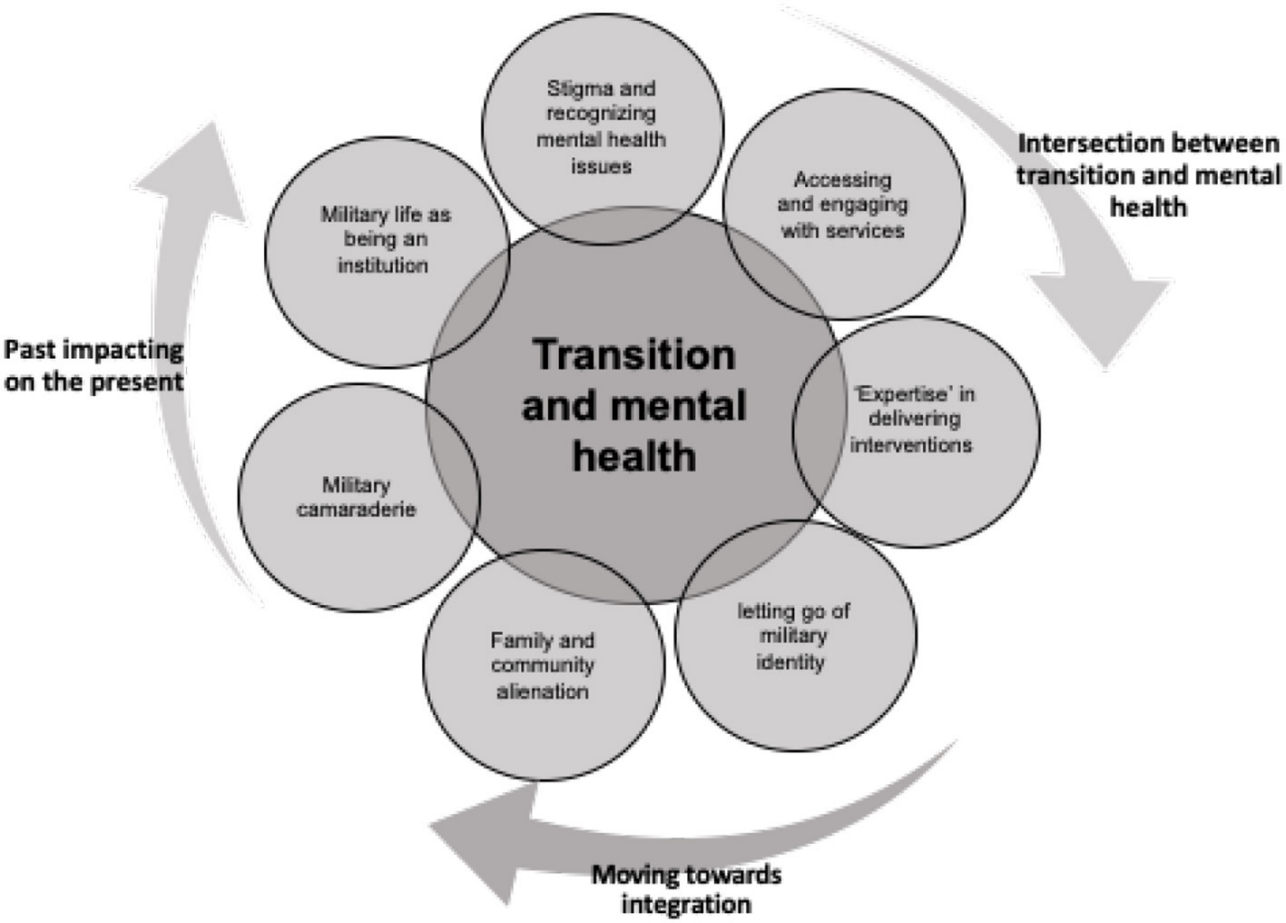
Seven interrelated themes related to the transition of the veterans into the community.

Seven interrelated sub-themes emerged specific to the internal processes surrounding the transition experience and related to constructs of how the veteran views, themselves, others and world, as illustrated in Figure 2:

1. Stigma and recognizing mental health issues
2. Accessing and engaging with services
3. “Expertise' in delivering interventions
4. Military life as institutionalized /stunted growth
5. Military camaraderie vs. community alienation
6. Suppressing the military identity/letting go of military identity
7. Family and community alienation

**Theme A: Intersection(s) between transition and mental health**

**Subtheme 1: Stigma and recognizing mental health issues**

The data were generated in the context of veterans seeking help and/or being referred to mental health services. Therefore, mental health service provision is central to the veterans' experiences in their transition to “civvy street” and as such, the transitions were understandably more challenging, because of the intersection between mental health and transition out of active service. This is vividly captured in the participants' accounts of their transitions as well as by the service lead.

It is acknowledged that mental health stigma associated with military culture could influence veterans' engagement with mental health services. This has been acknowledged by the service lead, i.e.,: that the veteran may not recognize themselves as having a mental health problem, which could lead to further delays in engaging with services:

“*[accessing the service is] fairly complex first of all, you know, that veteran might not feel that their mental health has been affected by their [military] service”*.

Moreover, the service lead highlighted that the word “veteran” itself may deter younger individuals from identifying as such and thus leading to delays in accessing services:

“*[the] word veteran itself has machinations and basically we identify [that] the younger guys don't like it, aren't too keen to be called veterans”*.

This echoes other findings from the UK (Burdett et al., 2013) which highlight disparities in the meaning of the term veteran for ex-service members, the official UK government definition and the public perceptions, which tend to focus on older veterans. Definitions of identity are important not only to the person holding them, but also to policy makers in providing provisions, and to the public with respect to social inclusion/exclusion; and discrepancies between these may materialize as barriers to engaging with support.

**Subtheme 2: Accessing and engaging with services**

There was a recognition that veterans will access general health services—such as General Practitioners (GPs)—and mostly rely on these professionals to recognize any veteran specific needs, which is not always the case. For example, the service lead saw themself as a signpost, acting as a mediator between the veteran, mental health and other services.

“*I'm bridging that gap and saying look go and see these people … who will be able to help you with your hearing … is a significant…relationship”*.

The expertise of the service provider / clinical staff was also important to the veteran. The veterans participants highlighted the clinical staff having personal and professional qualities that help service users feel at ease, as the quote below illustrates:

“*[What was most beneficial to you?] I think personalities, and the two people that I've seen are very welcoming, not judgmental at all, very understanding”*.

This suggests that clinical staff's non-judgmental and understanding attitude toward veterans is an important catalyst in engaging with the service. Furthermore, besides the qualities of the professional and the therapeutic relationship, there was a clear high regard for the military experience of the clinical staff and respect for staff coming from a military background:

“*You always look favorably because you know what they've done and you know what assets they can bring to a certain role”*.

These findings have potential implications for service development and delivery, suggesting that the presence of mental health professionals with military backgrounds, particularly at the access point, helps and/or speeds up veterans' engagement with the service. In the actual delivery of services and interventions, qualified mental health professionals' skills and abilities have a prominent role in maintaining the veterans' engagement. Nevertheless, the success of the intervention is undoubtedly augmented by the commitment of mental health professionals to develop and acquire knowledge and experience of working with veterans, which in turn will maintain and/or increase the effectiveness of the service.

The individual outcomes for the veterans were central messages deriving from most of the interviews, such as an improvement in their psychological wellbeing, from both their and their family and friends' perspective.

“*Well since I've had my sessions with [mental health professional] yes I mean you know before I was like a bottle of pop and I would have probably left by now”*.

**Subtheme 3: “Expertise” in delivering interventions**

Language appears to play a role in being understood, where there exists an intrinsic understanding between the veteran and mental health professional. This could also be an unspoken shared understanding that connects individuals and requires no further explanation. For example, one interviewee used the word “kinetic”, which contrary to shared meaning, was identified as a military term for destroying the enemy and objects associated with the enemy. Such recognition of a lived experience appears to validate the role of the mental health professional. Where a lack of military expertise exists, unintended consequences could occur, such as having to provide detailed explanation of local service protocols or withholding relevant information, leading to a sense of isolation.

The following quote speaks powerfully about the importance of the professional's veteran knowledge in addition to having mental health expertise:

“*I don't have to sugar coat it […] I don't have to explain acronyms […] I feel that I can consequently talk to [service professional] more freely than I can talk to other mental health professionals because […] the knowledge that he has been there, that he has worn the green skin and he knows what is like […] so I suppose you need somebody who is a veteran and an expert in the subject”*.

This indicates a recognition of shared identity as being an important aspect of communication. This could be what is verbalized and what is tacit knowledge about the direct experience of being a veteran. Therefore, a shared understanding is important in the transition from military to civilian life.

“*where you say something like that (to someone) that's never been in the army they just...they can't relate to it”*.

Here the interviewee has noted how being understood plays an important role in engagement with mental health services. The meaning of being understood appears to focus on having also been a veteran and therefore having insight into the role of active service personnel.

One participant discussed however how their GP made an effort to understand and research aspects of the military to better prepare for helping them:

“*[…] 12 months ago, there was a new GP came to our practice. Erm and I was having problems, quite by chance I ended up seeing him and he is extremely supportive. He [the GP] was asking, you know, can you get me some more information [about veterans' issues] and I said there you go. And he's very much gone away and, you know, done some research on it […] he's willing to learn more about it”*.

This finding highlights an important implication for practice, the need to raise awareness by heath care professionals about the special circumstances of veterans as this, in turn, has potential to impact positively on veterans' engagement with GP services.

However, the suggestion that veterans can only be effectively treated by veterans is not feasible or desirable, but could create barriers, as the service lead explained:

“*we can all fall into the trap of saying just because I served all veterans need to come and see someone who has served”*.

It is evident that having a shared understanding of contextual experiences is important to veterans. Such an understanding could include tacit knowledge of military service and thus veterans are more likely to talk openly (Jain et al., 2012; Hundt et al., 2015). It is not known how many active service personnel and /or veterans are trained and currently practice as mental health professionals. As such this cannot be relied on and may not be sustainable as a primary model of delivering mental health interventions. Although, as an alternative there is growing research evidence to suggest that veterans prefer peer support as part of transitioning to civilian life (Hundt et al., 2015; Drebing et al., 2018). This has also been recognized as playing an important part of clinical interventions, including both the encouragement to engage in services and also adherence to psycho-social interventions (Chinman et al., 2006; Hundt et al., 2015). Moreover, in light of emerging evidence (reviewed in Gettings et al., 2022) to indicate the need for an integrated system of interventions that encompasses peer support, it is crucial that service provision and development involves input from “experts by experience”.

**Theme B: Past interfering into present—“the military baggage” spilling into the transition**

Interviews reflected on aspects of the past military life that participants felt were still “spilling” into their transition, acting as barriers. “Military baggage” could therefore be defined as experiences gained from the military that have an impact on the transition into civilian life.

**Subtheme 4: Military life as institutionalized /stunted growth—negatives**

Some participants discussed how they viewed and perceived their transitions in powerful terms evoking ideas of institutionalized life, as one explains:

“*It's very difficult because I think… em … sounds a bit dramatic but I think you get institutionalized because someone is telling you what to do 24/7 where to be what clothes to wear and basically run your life and then you come out [of active service]. I mean that freedom of that is great, but then you realize that you have lost or not lost, but you lose touch with all your [military] friends and associates that you have built up in that career”*.

The word institutionalized implies that veterans have become accustomed to certain cultural norms in their military lives which are different to those they have come to experience on entering civilian life. This can cause difficulty in adjusting to the transition, especially the experience of displacement and subsequent isolation.

Some participants viewed their transition as a prompt to begin growing up:

“*If I'm honest I had not grown up within the MOD I was stuck in a routine my predominant behavior was like that of a 17 year old […] that was all that mattered. No big plans for the future no career it was all just live for today and it wasn't until probably after two years in civilian life where I felt yeah perhaps it is time to grow up and behave like an adult and think about tomorrow, as opposed to just today”*.

Kelty et al. (2010) pose that whilst the transition to adulthood may have been paused when services relied on conscription, since the introduction of entirely voluntary service personnel, the transition to adulthood is more stable and orderly for military servicemen than their civilian peers. This quote however implies that, due to the 'sheltered' lifestyle of the military, it was difficult to mature and begin planning for the future.

This concurs with the experience of many service men and women being recruited at a young age and who are voluntarily released from service before standard retirement age (The Howard League, 2011). The minimum age for enlisting in the UK Armed Forces is 16, although members of the Armed Forces cannot legally be deployed on the frontline until they turn 18. The recruitment of minors has been criticized by the United Nations Committee on the Rights of the Child, Parliament's own Joint Committee on Human Rights, and children's charities amongst others.

“*The UK is the only country in Europe and one of only 20 countries in the world to recruit 16-year-olds; these countries include no other member of NATO and no other permanent member of the UN Security Council. But they do include several regimes with little respect for human rights, including Iran, Zimbabwe and North Korea. (The Howard League*, *2011**, p. 8)”*.

**Subtheme 5: Military camaraderie vs. community alienation**

This subtheme links to an acknowledgment of the connections the veteran had whilst they were in service and the links they continue to have since their discharge from the military. The connection between service personnel appears to be fundamental to their experience whilst in service:

“*I mean the camaraderie is there and especially when you are at sea you live with the same people 24/7 because there is nowhere to go”*.

“*And it was the social side, it was the comradery of the military and they, we're very much part [of the military]”*.

The participants' acknowledgment of its absence during and after transition to the community leading to feeling isolated and a sense of alienation from the civilian community:

“*there is no camaraderie, well I say no camaraderie there is going to be some obviously but not like the forces”*.

‘*Because I have moved away from the like-minded people I don't feel I have that kind of bond with anybody.'*

Such contrasting experiences could lead to the formation of dichotomies, such as creating separation between themselves and others. Also the perception of different work ethic between civilian and veterans, in addition to the lack of connection with others leading to isolation. Such difference could infer that the work ethic of the veteran is superior to civilians, creating hostility.

“*Not on same wavelength [civilians] …Trivial …Lack of work ethic – civilians wait for someone else to do it”*.

Being misunderstood was also recognized as a barrier, one veteran commented on how civilians hold misconceptions about the military. Such constructs could be based on beliefs of what military life is like and focus on one aspect e.g., use of weapons and warfare. Therefore, is important to acknowledge how the veteran is perceived outside of the military.

**Theme C: Moving on, towards integration**

**Subtheme 6. Suppressing/letting go of military identity**

Several factors may impact on the formation of support relationships for veterans. For example, participants in this study expressed that they often felt they had to be mindful when speaking to civilians, as they may not understand them or feel comfortable around them. This can affect the formation of supportive relationships and lead to a sense of isolation:

“*You couldn't just come out with it down at the pub, […] you don't bother talking to people who haven't experienced these things”*.

“*[…] there I felt a great pressure to reign in who I was with my work colleagues because I didn't feel comfortable to be my natural self around people”*.

Some participants felt as if they had to suppress—or “reign in”—a part of their personality in order to fit in the civilian life rather than being themselves. As a consequence, creating a state of hypervigilance to their surroundings.

“*It felt frustrating and soul destroying […] that I was constantly on guard because I was aware there were times where I would delve back to who I was within the military”*.

Having to hide or change their identity, could encourage a type of hatred of self, “soul destroying”, adding to existing difficulties associated with the transition to civilian life. This could lead to the development of new and exacerbate existing mental health difficulties.

**Subtheme 7: Family and community alienation**

The role of family and friends in the transition to the community is apparent in the interviews. Participants discussed how relationships with family were sometimes difficult because they could not understand the lifestyle. One participant discussed how she chose to keep herself isolated from her family and not talk to them rather than try to explain:

“*You have a couple of close friends going on who I knew that I could talk to but I kept everyone else at a distance. Even for a lot of the time, my own family. It was just easier to avoid them than try and explain things”*.

Two veterans discussed how being married to an active service member meant connectivity to the military was maintained, therefore, recognizing the importance of being in contact with military life during their transition to the community:

“*I felt very lucky in that for personal reasons I married an RAF officer who was still serving when I got out so I stayed within the military community”*.

In addition, it was noted by three of the interviewees how the social aspects of the military are carried into civilian life. This may include engaging in civilian community activities, in attempt to recreate existing or establish new social connections:

“*And it was the social side, it was the comradery of the military and they, we're very much part of our local rugby club and still are, they have reunions every year. So it's getting back in touch with those people again”*.

Although it was noted by one veteran that some behaviors that were an acceptable part of service, became unhelpful when continued into civilian life:

“*Drinking with a crowd of people that was [drinking that carried on after leaving the army].”*

Therefore, there is a need to consider how such connections with others can be established in the transition from service to civilian life. The service lead acknowledged a myriad of difficulties the veteran may experience which included alcohol misuse. The use of peer mentoring as a way to support the transition was mentioned:

“*a strong social driver, peer support recovery mechanism”*.

Finally, all of the interviewees, including the service lead noted how serving in the military impacted on their psychological and physical wellbeing. Also, the positive growth this can create through learning other coping strategies in their transition to civilian life. It is evident that this shaped how they viewed their transition into the community, including one veteran who highlighted regret in joining the military.

## 6. Discussions and implications for clinical settings

**Figure 3:**
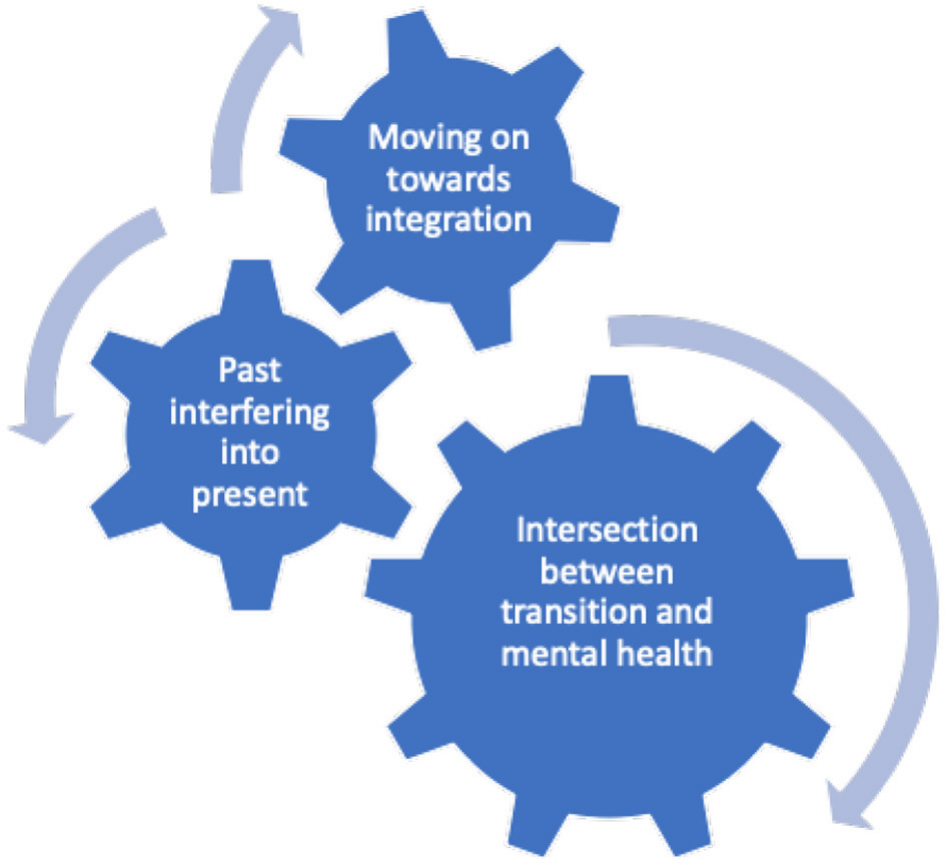
The interconnected themes identified in the transition experience of veterans returning to the community.

From data presented above, it is evident that there are a variety of factors that influence the experience a veteran has when returning to the civilian community. Three distinctive overarching themes emerged from the research; intersection between transition and mental health; past interfering into present; moving on toward integration (see Figure 3).

Some of these factors were identified as barriers to the transition to civilian life and subsequently negatively impact on the veteran's wellbeing.

### 6.1. Barriers to accessing services

The findings identify various barriers both in accessing and engaging with the community services, which appears to be fundamental in influencing their perception of transition from the military. There is much existing research that recognizes the lack of accessible services for veterans to engage with in the community. This could relate to the availability of a particular service e.g., waiting list a lack of awareness of a service existence. In addition, not knowing the right questions to ask or indeed not being able to recognize or verbalize difficulties they may be experiencing can also act as a barrier to accessing service. The perception of engaging with services also plays a role in decision making. It is evident that this can negatively impact on the veteran's wellbeing (Wands, 2013; Ahern et al., 2015; Oster et al., 2017).

The research has highlighted some common themes that influence the veteran's decision to access services or not, that concurs with existing research. This includes the decision to access all sources of help, including psychological therapy. These themes include the stigma around mental health, where the veteran sees themselves or that others perceive them as being weak (Rafferty et al., 2018; Mark et al., 2019). The lack of understanding about what mental health is and a focus on physical health problems could lead to a delay in diagnosis or misdiagnosis. The veteran may not recognize or see themselves as needing help and thus hold a reluctance to engage in formal treatment such as regular group or individual therapies, instead choosing informal interventions (Stevelink et al., 2018; Mark et al., 2019), although there is evidence to suggest that peer support can be an effective intervention (Hundt et al., 2015; Drebing et al., 2018). Interestingly, many of these identified themes—such as mental health stigma, lack of understanding/recognizing of own mental health difficultie—could also relate to the general population (Mark et al., 2019).

### 6.2. Moral Injury vs. PTSD

The way veterans see themselves appears to play an important role in transitioning to civilian life. The emerging construct specific to *moral injury* could offer a way to understand the inner experiences of veterans (Williamson et al., 2021a). Litz et al. (2009) considers this as the aftermath of being faced with ethical dilemmas such as witnessing rather than preventing, taking part in acts that lead to death or injury. Although these acts may be the rules of engagement, they may transgress core beliefs held by the veteran. Specifically, the self-constructs that result from events experienced whist actively serving in a war zone, may present as challenge to their sense of humanity and result in feelings of guilt or shame (Litz et al., 2009; Currier et al., 2015; Williamson et al., 2021a). This can increase the complexity of the presentation difficulties e.g., complex trauma. By gaining an insight into the individual's conceptualization of moral injury it is proposed that more effective evidence-based interventions can be implemented to address the wide-ranging needs of veterans (Litz et al., 2009; Currier et al., 2015). As such services can work with the veteran to “heal” the moral injury (Williamson et al., 2021b).

Diagnostic criteria specific to PTSD has only recently referred to moral challenge as a relevant factor in the type of exposure event (Nash, 2019; Borges et al., 2022; Phelps et al., 2022). Although arguably it only remains relevant to diagnostic criteria, if they result from actual or threatened death, serious injury and/or sexual violence (Williamson et al., 2021a; Borges et al., 2022; Phelps et al., 2022). In the context of PTSD, cognitive behavioral therapy approaches argue that trauma related cognitions have become exaggerated (Farnsworth, 2019). This may unintentionally lead to stigmatization as the individual is inaccurately appraising the situation (Nash, 2019). Whereas, a moral injury approach argues the individual adopts incorrect self-beliefs specific to culpability, leading to avoidance of painful moral emotions such as guilt and shame (Williamson et al., 2021b). The recognition of moral injury supports moves toward using third wave therapies, such as acceptance commitment therapy to enable veterans to live alongside their values whilst experiencing moral pain (Borges et al., 2022). This approach shifts away from traditional cognitive behavioral approaches of prolonged exposure and Eye Movement Desensitization and Reprocessing (EMDR) (Borges et al., 2022; Phelps et al., 2022).

### 6.3. Theories underpinning the transition to the community

Of particular note is homecoming theory that is consistent with these findings as it suggests there is a discrepancy between the veteran's expectations and the actual experience encountered (Borus, 1975; Faulkner and McGaw, 1977). Such a mismatch could relate to difference encountered in the military services and changes the veteran has undergone, perhaps through the experiences encountered whilst serving—for example, changes brought by combat injuries. Here cultural differences are noted between the military and non-military service provision (Koo et al., 2015). More specifically a sense of difference increases the probability of veterans using unhelpful behaviors which then impact on the transition and their general wellbeing (Ahern et al., 2015; Oster et al., 2017; Derefinko et al., 2018). Such unhelpful or risk-taking behaviors could include the use of substances (Pease et al., 2016). Research conducted by Derefinko et al. (2018) indicates the need for interventions specific to substances use and psychological wellbeing, before and after the transition. Once entering civilian services, if the professionals lack knowledge specific to veteran needs, delays could occur in accessing the most appropriate service. Most of the interviewees inferred that they had experienced crisis points in the experience of their difficulties, in particular mental health. Arguably this could have been prevented had support services been in place coupled with an increased awareness of the specific physical and mental health needs of veterans.

### 6.4. The role of the MOD^1^ in the UK context

A review of veteran's services highlighted the importance of the role of the Ministry of Defence (MOD) in challenging perceptions of veterans and its wider responsibilities to all veteran's (MOD, 2023). Thus, such issues are addressed by the military, as part of supporting military personnel transition to civilian life. These acts of preparedness occur at the start of an individual's military career. Such a process may miss certain veterans, for example those who have been dishonorably discharged. Therefore, there is opportunity to ensure that basic processes are in place at the transition point, for example, supporting veterans to register with a local General Practitioner (GP) surgery in their community, who often acts as a gateway for onward referral to specialist services.

A general lack of information about civilian services available was noted. There was one exception of a veteran who was discharged from the military-to-military prison where he served a 6-month sentence. Although this would have presented challenges, equally this may have enabled easier access to support services and aiding transition to the community (Fazel and Baillargeon, 2010; Wainwright et al., 2017). This includes support within the prison and referral to services within the community, on being released. Research also highlighted the importance of peer support holding value in enabling veterans to reengage with veteran services after a prison stay (Simmons et al., 2017). There are apparent regional variations of such service provision potentially creates inequity (Phillips, 2014). As well as delays being incurred, there may be the relative absence of services, unless the veteran can travel. Although the Armed Forces Covenant recognizes the distinctive healthcare needs veterans have and the necessity for priority access (MOD Defence Holistic Transition Policy, 2019). This is facilitated by the NHS Constitution which clearly defines active service personnel and their families to ensure they are not disadvantaged (Department of Health, 2015). However, it is evident that there are additional barriers to accessing such services, such as having a lack of knowledge of veteran specific needs (Iversen et al., 2005; Wainwright et al., 2017). It is important to note that at the time of writing, 104 of 219 NHS Trusts are accredited as exemplars of best practice for veterans and their families (NHS England, 2022). The findings of this research support the Armed Forces Covenant recommendation that veterans should have access to mental health professionals who hold an understanding of military culture (MOD Defence Holistic Transition Policy, 2019). Therefore, there is a need to consider how to commission services in a consistent and accessible manner.

### 6.5. The “military family”

One consistent theme was that of identification with the view of the military as family. Each veteran highlights the sense of shared identity during active service and the contrast of its absence on discharge. Also, the sense of belonging and identification with other veterans. Such a unique sense of belonging was created in service and acted to reinforce connectedness to other service personnel (Libin et al., 2017). This is supported by social climate theory that highlights specific characteristics that underpin an organization such as the military (Moos, 2002; Ahern et al., 2015). Equally this had a conflicting effect as it could serves to disconnect from family and friends outside of the military (Libin et al., 2017). There appears a juxtaposition between comradery between service personnel and the consequence of institutionalization, where the former constructs the latter. Whilst this calls for support for family and friends of the service personal, it also highlights the need for peer mentoring (Williamson et al., 2021c). Research has found that former service personnel who engaged with peer mentoring have successfully adjusted and reintegrated to civilian life (Greden et al., 2010; Repper and Carter, 2011; Pfeiffer et al., 2012). Ahern et al. (2015) recognizes this as key resource in a successful transition and call this individual a peer navigator, an experienced veteran who provides advice and support. Support could also help to reduce a sense of isolation through promoting engagement with the community. Such connection with a peer could help manage any barriers and facilitate engagement with other services. This approach could help to reduce veterans experiencing crises and act to reduce hospital admissions (Repper and Carter, 2011). However, as one interviewee stated peer interactions should not act to prevent or delay interaction with the community, rather it should act to enable the journey. There is a growing body of research regarding peer led service provision in both the veteran and civilian population (Cheney et al., 2016; Shaw et al., 2021; Anderson et al., 2022; Gettings et al., 2022). Although such peer interventions hold great value there is a lack of research into their effectiveness (Ahern et al., 2015; Derefinko et al., 2018).

### 6.6. The importance of “perception”

There was also a lack of mental health awareness noted by three of the veterans, that included misinformation about mental health diagnoses. This could relate to stigma and prejudice with which it is often associated. One interviewee in particular noted their detainment in military prison as being a consequence of them experiencing symptoms of post-traumatic stress disorder (Wainwright et al., 2016). Such an awareness of mental health should operate both in the military and within the community. Whilst this could reflect a disparity in knowledge of mental health within the military, it could also reflect a paradigm shift at a societal level, where there is a superficial knowledge of mental health diagnosis, however, it lacks the necessary details. In adopting such a reductionist approach the whole person approach is neglected and individuals become marginalized with mental health labels. One clear perception is that veterans only face difficulties with post-traumatic stress disorder (Ahern et al., 2015; Oster et al., 2017; Derefinko et al., 2018). Such marginalization could subsequently be viewed as stigma both within the military and in the community and restrict access to services (Oster et al., 2017). This concurs with dichotomous constructs that are evident in many of the veteran's narrative. For example, the inference of having an earlier mental health diagnosis would have prevented the adoption of unhelpful behaviors or unintended consequences (Derefinko et al., 2018). This may have avoided difficulties encountered in the transition to civilian life, including the breakdown of relationships, substance misuse and loss of civilian careers. This highlights a training need to educate healthcare service providers in the specific needs of veterans and their families.

## 7. Conclusions

Much of the research cited in support of arguments made in this article is largely from the United States of America, reflective of current military activity in recent areas of conflict. Whilst this research is directly applicable to the United Kingdom it is important to note socio-economic differences exist between the two countries. This does, however, highlight the need for more research to be conducted specific to veterans in the United Kingdom. Our research indicates that the support infrastructure put in place for veterans is essential to ease the complexities surrounding the transition from the military to civilian life. Such transition support includes both the military and civilian communities alike, for example, peer to peer interaction to assist in the navigation of accessing civilian life (Ahern et al., 2015). The sense of veteran isolation is apparent and fostering ways to reconnect with family and friends after periods of absence is essential (Williamson et al., 2021c). Challenging the stigma of mental health is also of paramount importance, as well as the perception of veterans, once they are in the community. There is also a need for highlighting the awareness of veteran specific needs both within the wider community and in non-military-based services. This could be conducted through integrating veteran specific information into existing training or through the development of new training programmes.

The importance of preparing for transition at all stages is also highlighted, from being in, to leaving the military, and essential to the wellbeing of veterans (Black et al., 2007; Oster et al., 2017). The Active Service personnel of today are the veterans of the future. Over half of them will have partners who also face the transition to civilian life. While the term “transition” is used by the Armed Forces to describe the period of integration from service life to civilian life, the MOD Transition Policy (MOD, 2017) takes a “transition through life” approach. Such an approach should enable preparation for leaving the military to begin much earlier in a military career, especially as Serving personnel and their families face multiple transitions from the moment they join the Armed Forces until the day they leave. If these transitions are handled well, they can build resilience for the ultimate transition into “civvy street” (Walker et al., 2020). Inevitably, as we have seen, the shared identity of members of the military creates a sense of “family” which continues long after leaving the military. This is often fostered by veteran organizations who keep the identity alive.

Leaving the military at the end of a career can invoke a range of emotions which can include anxiety, a strong sense of loss, and worries about mental health issues. Since civilians can face long waiting lists for mental health support it is essential that personnel with mental health concerns, including PTSD, are enabled to transfer to community-based services seamlessly and speedily. Therefore, for a successful transition, a whole person approach is needed, within an integrated care system. The MOD Defence Holistic Transition Policy (2019) promotes this holistic support, and the UK Strategy for Our Veterans (2018)^2^ encompasses a 10-year vision to ensure a smooth transition to military life. Appropriate support must therefore be in place throughout a person's military career and long before the moment of transition to civilian life. Also, because families often hold the key to a positive transition, involving them in the transition process is vital. Two recommendations from the ‘Living in Our Shoes review (Walker et al., 2020) are particularly salient here. These focused on the importance of transition and resettlement pathways always including family members; and the need for a joined-up approach across government, local authorities, military charities, and the private sectors to provide consistent and seamless resettlement processes for all service leavers and their families.

### 7.1. Clinical practice implications

In the context of moral injury, both homecoming theory and transition theory offer effective ways for clinicians to support veteran transition into civilian life. Homecoming theory is highly relevant in the context of interventions that target the disconnection and alienation that can occur. In the knowledge that cognitive behavioral approaches may contribute to the mental health stigma there is a call to shift away from the traditional cognitive behavioral approaches towards third wave therapies, such as Acceptance Commitment Therapy (ACT).

### 7.2. Organizational implications

Our study contributes to the evidence base informing the development of effective pathways into Mental Health Services for UK Veterans (Osborne et al., 2021). There is also a need to consider how organizations can move toward supporting veteran's transition, through organizational design. Schlossberg's transition theory provides a framework to assess the organizations readiness to support the transition of veterans from the military. This coupled with appreciative inquiry could then create change to improve veteran outcomes.

### 7.3. Research implications

This study offers an insight into the lived experiences of a sample of veterans attending a mental health support service in the UK. Such in-depth narrative data, albeit coming from a small sample of veterans in this study, are essential to support the development of further research with a larger cohort of participants. More research on larger and diverse groups of veterans is crucially needed to enhance understanding of how to facilitate their transition and develop evidence-based interventions to support their transitions, including reconnection with family and the broader community, while preserving military-peer connections too.

## Data availability statement

The raw data supporting the conclusions of this article will be made available by the authors, without undue reservation.

## Ethics statement

The studies involving human participants were reviewed and approved by University of Worcester, Institute of Health and Society Research Ethics Committee. The patients/participants provided their informed consent to participate in this study.

## Author contributions

GM designed and supervised data collection and analysis, writing up and drafting of the paper, and principal investigator of the original study. JA re-analyzed data and drafted the paper. JR contributed to original data collection, analysis, and writing up of findings. JW advised on the practice and policy implications. All authors have contributed to, reviewed, and approved the final manuscript.

## Footnotes

^1^UK Ministry of Defence (MOD).

^2^Veterans' Transition Review. Available at: https://www.gov.uk/government/news/veterans-transition-review-has-been-published (accessed May 13, 2018).
